# Role of Hog1-mediated stress tolerance in biofilm formation by the pathogenic fungus *Trichosporon asahii*

**DOI:** 10.1038/s41598-024-80200-z

**Published:** 2024-11-20

**Authors:** Yasuhiko Matsumoto, Mei Nakayama, Yuta Shimizu, Sachi Koganesawa, Hiromi Kanai, Yu Sugiyama, Sanae Kurakado, Takashi Sugita

**Affiliations:** Department of Microbiology, Meiji Pharmaceutical University, 2-522-1, Noshio, Kiyose, Tokyo 204-8588 Japan

**Keywords:** *Trichosporon asahii*, Biofilm, Mitogen-activated protein kinase, Stress tolerance, Infection, Biofilms, Fungi, Eukaryote, Experimental models of disease, Genetics research, Cell growth

## Abstract

*Trichosporon asahii*, a dimorphic fungus, causes bloodstream infections in immunocompromised patients with neutropenia*.* Biofilms are formed on the surfaces of medical devices such as catheters as *T. asahii* transitions morphologically from yeast to hyphae in the host environment. Oxidative stress tolerance and morphological changes of *T. asahii* are regulated by Hog1, a mitogen-activated protein kinase. The role of Hog1 in the biofilm formation by *T. asahii,* however, has remained unknown. In the present study, we demonstrated that a *hog1* gene-deficient *T. asahii* mutant formed excess biofilm under a rich medium in vitro, but did not form biofilm in an in vivo evaluation system using silkworms. The *hog1* gene-deficient *T. asahii* mutant formed a greater amount of biofilm than the parent strain in vitro. Under an oxidative stress condition in vitro, however, lower amounts of biofilm were formed by the *hog1* gene-deficient *T. asahii* mutant than by the parent strain. In an in vivo evaluation system using silkworms, lower amounts of biofilm were formed by the *hog1* gene-deficient *T. asahii* mutant than by the parent strain. Our findings suggest that Hog1 regulates biofilm formation by *T. asahii* in response to host environmental conditions, including oxidative stress.

## Introduction

The pathogenic fungus *Trichosporon asahii* exhibits several morphological forms, including yeast, hyphae, and arthroconidia^1^, and can be isolated from various sources, including soil and human samples such as blood, sputum, skin, feces, and urine^2–4^. In immunocompromised patients with neutropenia, *T. asahii* causes severe bloodstream infections, such as catheter-related fungemia^5,6^. In catheter-related fungemia, *T. asahii* forms a biofilm comprising cells and an extracellular matrix of polysaccharides, proteins, nucleic acids, and lipids on the surface of the catheter^7,8^. The morphology of *T. asahii* is related to its biofilm formation^1,7^. *T. asahii* in biofilm is resistant to treatment with ethanol and antifungal drugs, such as amphotericin B, caspofungin, and voriconazole^7,9^. The development of effective preventive methods against catheter-related bloodstream infections by *T. asahii* requires a deeper understanding of the molecular mechanisms underlying biofilm formation by *T. asahii*.

Hog1, a mitogen-activated protein kinase (MAPK), is involved in fungal resistance to several types of stressors^10–12^. The Hog1-mediated signaling pathway regulates the expression of genes related to stress resistance^10–12^. Hog1 is widely conserved in fungi and plays crucial roles in the resistance to oxidative stress in *T. asahii*, *Cryptococcus neoformans*, *Candida albicans*, and *Saccharomyces cerevisiae*^13–20^. Moreover, *hog1* gene deficiency causes excess hyphal formation in *T. asahii*^13^. A *hog1* gene deficiency in *T. asahii* leads to a higher proportion of the hyphal morphology^13^ and a lower proportion of the yeast and arthroconidia morphologies^13^, suggesting that Hog1 negatively regulates the transition of yeast to hyphae in *T. asahii*. On the other hand, the *hog1* gene-deficient *T. asahii* mutant exhibits sensitivity to oxidative stress, including H_2_O_2_ exposure, and displays an avirulent phenotype in a silkworm infection model^13^. Therefore, Hog1 is required for the oxidative stress tolerance and virulence of *T. asahii* against silkworms. The precise role of Hog1 in biofilm formation by *T. asahii,* however, has remained unknown.

Biofilm formation by the pathogenic fungus *C. albicans* on a catheter surface is affected by host environmental factors, such as nutrition, proteins, and interactions with host cells^21,22^. The catheter material-inserted silkworm infection model is useful for in vivo evaluation of biofilm formation by *C. albicans*^23–25^. A single fiber of polyurethane, the same material used to construct catheters, is inserted into the silkworm hemolymph under the skin surface^23^. *C. albicans* forms a biofilm on the fiber surface in the hemolymph^23,26^ and exhibits tolerance to antifungal drugs, such as amphotericin B, fluconazole, and voriconazole^23,24^, suggesting the potential utility of this model for evaluating in vivo biofilm formation by *T. asahii*.

In the present study, we found that the *hog1* gene-deficient *T. asahii* mutant forms excess biofilm with long hyphae in vitro. Under oxidative stress conditions, such as H_2_O_2_ exposure, less biofilm was formed by the *hog1* gene-deficient *T. asahii* mutant than by the parent strain in vitro. Moreover, hyphal formation in the biofilm formed by mutant was inhibited by H_2_O_2_ in vitro. In an in vivo evaluation system using the fiber-inserted silkworm infection model, *hog1* gene deficiency in *T. asahii* decreased the formation of biofilm on the polyurethane fiber surface. Our findings suggest that Hog1 is essential for biofilm formation by *T. asahii *in vivo, but dispensable under in vitro conditions without stress.

## Methods

### Reagents

Nourseothricin was purchased from Jena Bioscience (Dortmund, Germany). Cefotaxime sodium, D-glucose, agar, crystal violet, acetic acid, and H_2_O_2_ were purchased from Fujifilm Wako Pure Chemical Industries (Osaka, Japan). Hygromycin B was purchased from Tokyo Chemical Industry Co., Ltd. (Tokyo, Japan). G418 was purchased from Enzo Life Science, Inc. (Farmingdale, NY, USA). Hipolypeptone was purchased from Nihon Pharmaceutical Co., Ltd. (Tokyo, Japan). The 2,3-bis-(2-methoxy-4-nitro-5-sulfophenyl)-2H-tetrazolium-5-carboxanilide (XTT) was purchased from Biotium (Freemont, CA, USA). Calcofluor white (CFW) stain solution, menadione, and RPMI-1640 medium were purchased from Sigma-Aldrich (St. Louis, MO, USA).

### *Trichosporon asahii* cultures

The *T. asahii* strain (MPU129 *ku70* gene-deficient mutant) used in this study was generated as previously reported^27,28^. The *hog1* gene-deficient *T. asahii* mutant and complemented strain were also cultured as previously reported^13^. Information on the strains is provided in Table 1. Culture of *T. asahii* strains was performed according to a previous report^29^. The *T. asahii* strains were grown on Sabouraud dextrose agar (SDA; 1% hipolypeptone, 4% dextrose, and 1.5% agar) containing G418 (100 μg/ml) and incubated at 27 °C for 2 days.

**Table 1:** *T. asahii* strains used in this study.

*T. asahii* strains	Relevant genotype	Background	Reference
MPU129 ∆*ku70* (Parent strain)	*ku70*::*nptII*	MPU129	Matsumoto et. al.^27^
∆*hog1*	*ku70*::*nptII*, *hog1*::*NAT1*	MPU129 ∆*ku70*	Matsumoto et. al.^13^
Comp. (Complemented strain)	*ku70*::*nptII*, *NAT1*::*hog1*, *hph*	∆*hog1*	Matsumoto et. al.^13^

### Biofilm measurements in vitro

The biofilm formation assay was performed as described previously^1^. The *T. asahii* strains were grown on SDA at 27 °C for 2 days. The *T. asahii* cells were suspended in physiological saline solution and filtered through a 40-μm cell strainer (Corning Inc., Corning, NY, USA). The cell suspension was adjusted to an absorbance of 0.1 at 630 nm with Sabouraud medium or RPMI medium (RPMI 1640 with 3-(N-morpholino) propanesulfonic acid, pH 7.0). The filtered cell suspensions (100 µL) were applied to wells of 96-well microtiter plate (Techno Plastic Products, Trasadingen, Switzerland) and incubated at 37 °C for 1 h. After incubation, the supernatants were removed. The wells with *T. asahii* cells were washed with phosphate-buffered saline (PBS) and fresh medium was added. After incubation at 37 °C for 24 h, the supernatant was removed and replaced with fresh medium. After another 24-h incubation, planktonic cells were removed, and the wells were washed three times with PBS. In the case of H_2_O_2_ treatment, Sabouraud medium with H_2_O_2_ (5 mM) was used.

Biofilm mass was measured using crystal violet. The crystal violet solution (0.1%, 50 µL) was added to the dried wells and the plate was incubated for 30 min. After incubation, the wells were washed three times with PBS and dried for 30 min. Acetic acid solution (33%, 50 µL) was added to the wells and absorbance at 550 nm was measured using a microplate reader (iMark microplate reader; Bio-Rad Laboratories Inc., Hercules, CA, USA).

Cell viability in biofilm was measured using XTT. The XTT solution with 2 µM menadione (100 µL) was added to the wells and the plate was incubated at 37 °C for 1 h. After incubation, absorbance at 490 and 630 nm was measured using a microplate reader (iMark microplate reader; Bio-Rad Laboratories Inc.).

### Observation of *T. asahii* morphology in the biofilm

*T. asahii* cells were suspended in a physiological saline solution and filtered through a 40-μm cell strainer (Corning Inc.). The filtered cell suspension was adjusted to an absorbance of 0.1 at 630 nm using Sabouraud medium. The cell suspensions (100 µL) were applied to wells of a 96-well microtiter plate (Techno Plastic Products) and incubated at 37 °C for 1 h before removing the supernatants. The wells with *T. asahii* cells were washed with PBS and fresh medium was added. After incubation at 37 °C for 24 h, the supernatant was removed and replaced with fresh medium. After another 24-h incubation, planktonic cells were removed and the wells were washed three times with PBS. CFW stain solution (100 µL) was added to the wells and the plate was incubated at 25 °C for 15 min. The cells were observed and photographed under a fluorescence microscope (BZ-X800; Keyence Corporation, Osaka, Japan). The fluorescence of the images was determined by Image J software (Image J 1.47t; National Institutes of Health, Bethesda, MD).

### Biofilm assay in an in vivo system using silkworms

Silkworm infection experiments to observe biofilm formed by *T. asahii* in vivo were conducted as previously described with slight modifications^23,26^. Eggs of silkworms (KINSYU × SHOWA) were purchased from Ehime-Sanshu Co, Ltd. (Ehime, Japan). Silkworm fifth instar larvae were fed overnight with an artificial diet (Silkmate 2S; Ehime-Sanshu Co., Ltd.). A polyurethane fiber (0.5 mm thick, Gomutegusu F046, No. H3; Daiso-Sangyo, Hiroshima, Japan) was cut into 2-cm lengths, treated with a 70% ethanol solution for 15 min, and then dried under UV irradiation for 30 min. A small hole was punctured on the back of each silkworm using a marking pin (Daiso-Sangyo), and a UV-sterilized polyurethane fiber was then inserted under the skin surface into the silkworm hemolymph through the hole. The fiber-inserted silkworms were observed at 25 °C for 30 min to ensure that the bleeding stopped. *T. asahii* cells grown on SDA plates incubated for 1 day at 27 °C were suspended in physiological saline and filtered through a 40-μm cell strainer (Corning Inc.). The silkworms were injected with a 50-µL suspension of *T. asahii* cells (absorbance of 630 = 2) and incubated at 27 °C for 24 h. The polyurethane fibers recovered from the silkworms were transferred to a 1.5-ml tube, washed twice with saline, and treated with methanol for 20 min. After removing the methanol solution, the polyurethane fibers were dried for 1 h. A 0.1% (w/v) aqueous crystal violet solution (350 µL) was added to the tube and the tube was incubated at 25 °C for 20 min. After removing the staining solution, the polyurethane fibers were washed twice with 20% ethanol and once with distilled water. The biofilm on the polyurethane fiber surface was observed under a microscope (CH-30; Olympus, Tokyo, Japan). After microscopic observation, the polyurethane fibers were placed in 33% (v/v) acetic acid (500 µL) for 30 min and distilled water (500 µL) was added. The absorbance of each solution at A590 was measured.

### Statistical analysis

**Fig. 1:**
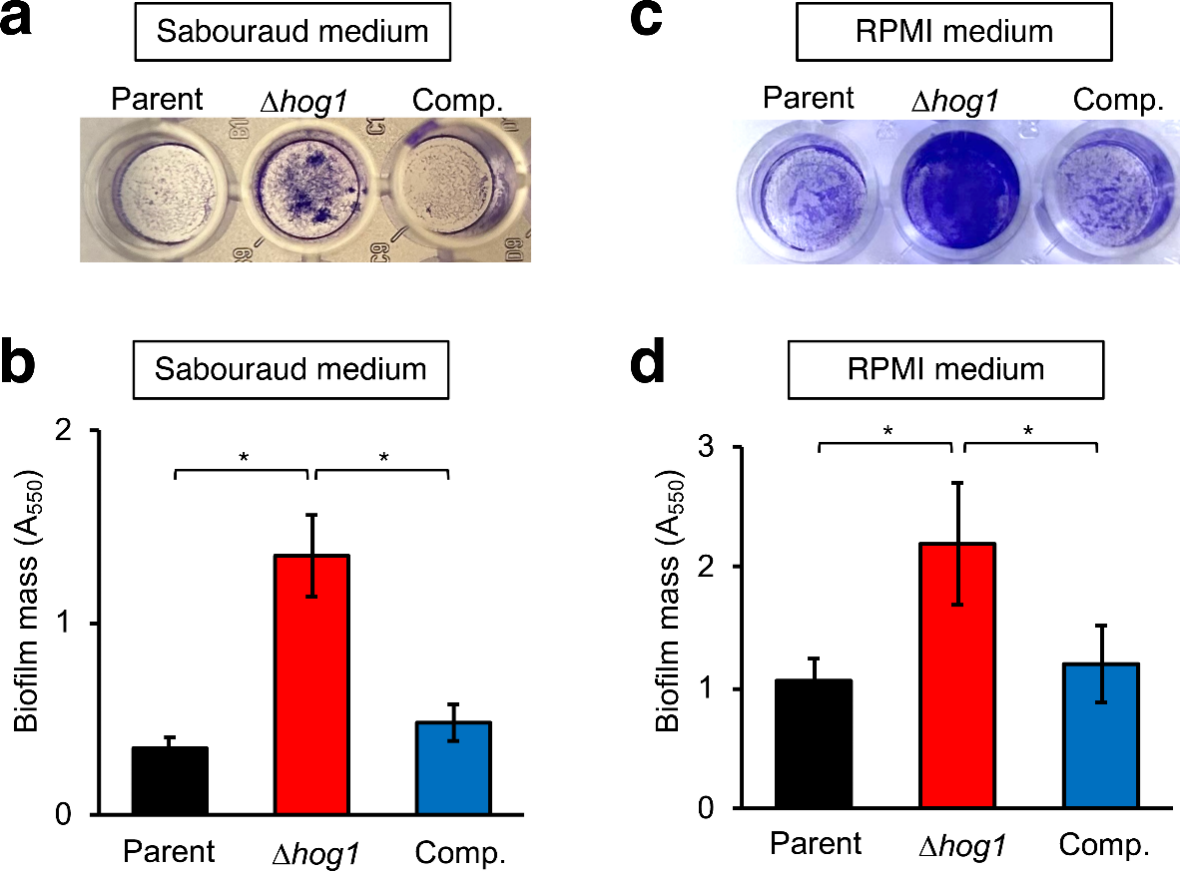
Excess biofilm formation by the *hog1* gene-deficient mutant of *T. asahii * in vitro. (**a**, **b**) Biofilm formation by *T. asahii* in Sabouraud dextrose medium in vitro was determined by crystal violet (CV) staining. The amounts of biofilm formed by the parent strain (Parent), *hog1* gene-deficient mutant (∆*hog1*), and its complemented strain (Comp.) in Sabouraud dextrose medium were determined by CV staining. (**c**, **d**) Biofilm formation by *T. asahii* in RPMI medium in vitro. The amounts of biofilm formation by the parent strain (Parent), *hog1* gene-deficient mutant (∆*hog1*), and its complemented strain (Comp.) in the RPMI medium were determined by CV staining. (**a**, **c**) Pictures of biofilm stained with CV are shown. (**b**, **d**) The amounts of CV were determined by measuring the absorbance at 550 nm (A_550_). Data are shown as means ± standard deviation (SD). Statistically significant differences between groups were evaluated using Tukey’s test. * *P* < 0.05. n = 5/group.

**Fig. 2:**
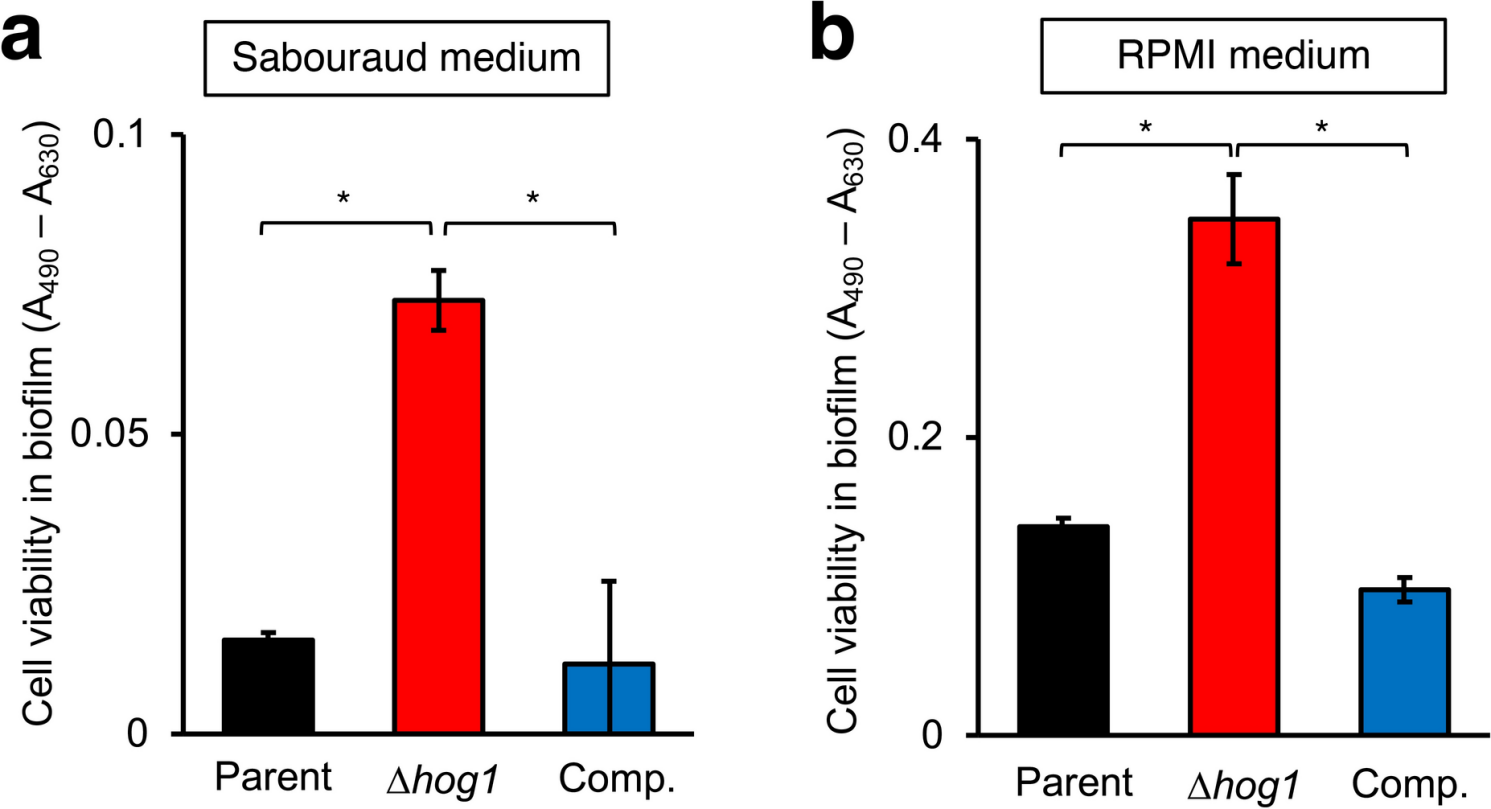
Increase in cell viability in the biofilm of the *hog1* gene-deficient mutant of *T. asahii* in vitro. Cell viability in *T. asahii* biofilm was determined by the XTT assay. Cell viabilities in the biofilm of the parent strain (Parent), *hog1* gene-deficient mutant (∆*hog1*), and its complemented strain (Comp.) in Sabouraud dextrose medium (**a**) or RPMI medium (**b**) were determined. The amounts of XTT were determined by measuring the absorbance at 490 (A_490_) and 630 nm (A_630_). Data are shown as means ± standard deviation (SD). Statistically significant differences between groups were evaluated using Tukey’s test. *P < 0.05. n = 3–5/group.

**Fig. 3:**
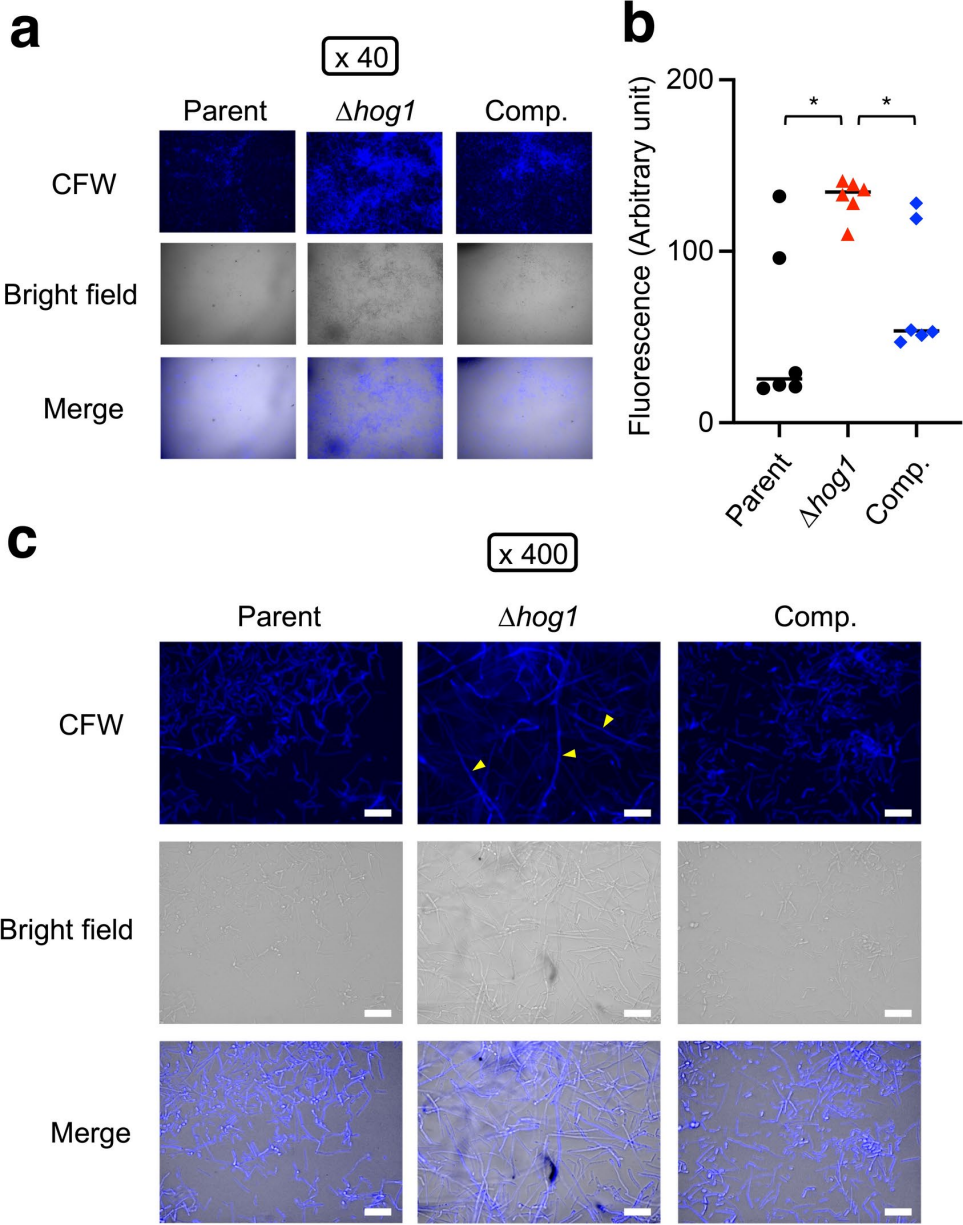
Hyphal formation of the *hog1* gene-deficient mutant in biofilm. The morphology of *T. asahii* stained with CFW was observed using fluorescence microscopy. The parent strain (Parent), the *hog1* gene-deficient mutant (∆*hog1*), and the complemented strain of ∆*hog1* (Comp.) in the biofilm were observed. CFW: calcofluor white stain. (**a**) Observed under a microscope at 40-fold magnification (×40). (**b**) Measurement of CFW fluorescence. Statistically significant differences between groups were evaluated using Tukey’s test. *P < 0.05. n = 6/group. (**c**) Observed under a microscope at 400-fold magnification (×400). White scale bar = 20 µm. Yellow arrows indicate long hyphae.

**Fig. 4:**
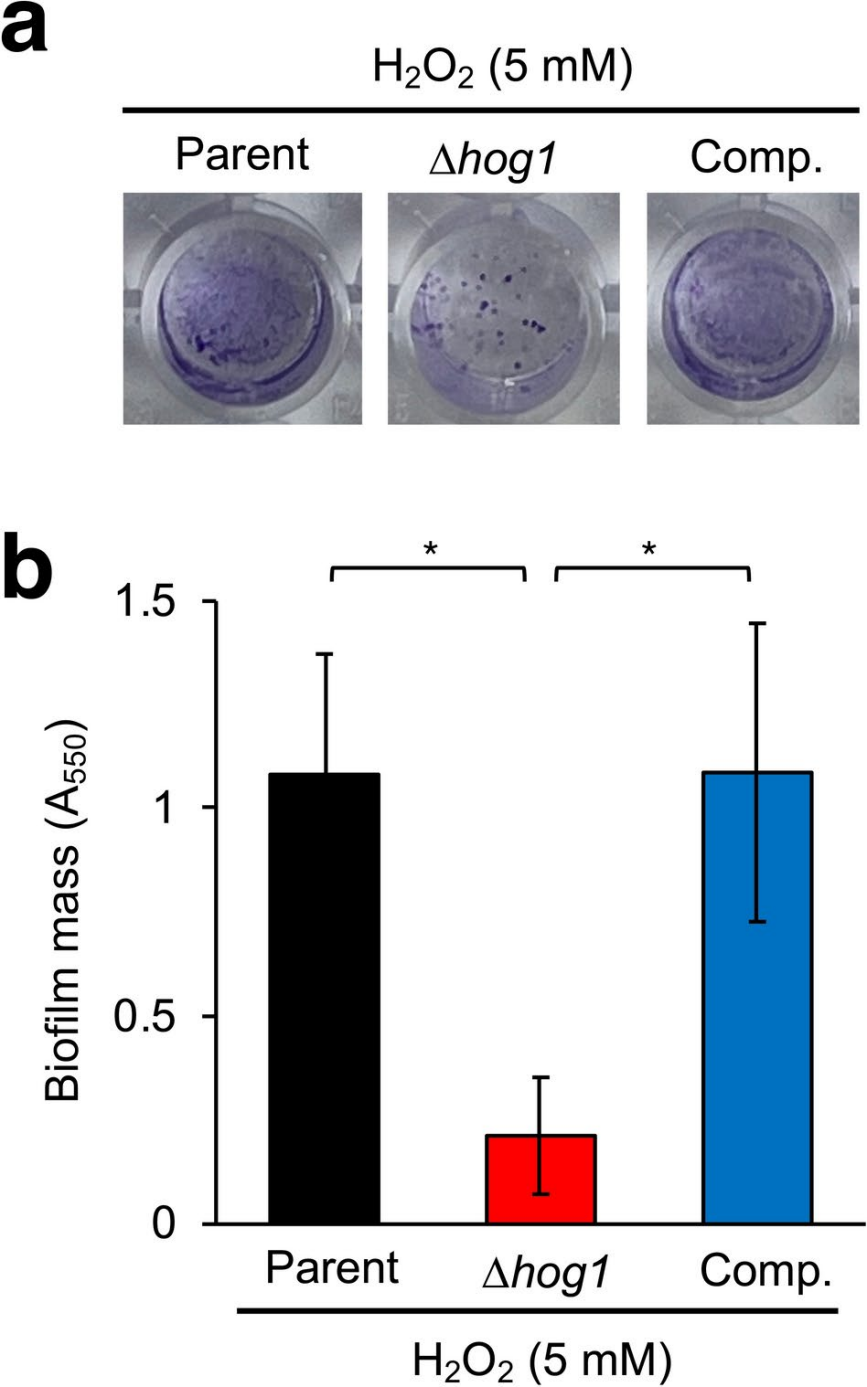
Effect of H_2_O_2_ on biofilm formation by the *hog1* gene-deficient mutant of *T. asahii* in vitro. (**a**, **b**) Biofilm formation by *T. asahii* in Sabouraud dextrose medium in vitro was determined by crystal violet (CV) staining. The amounts of biofilm formed by the parent strain (Parent), *hog1* gene-deficient mutant (∆*hog1*), and its complemented strain (Comp.) in Sabouraud dextrose medium were determined by CV staining. (**a**) Pictures of biofilm stained with CV are shown. (**b**) The amounts of CV were determined by measuring the absorbance at 550 nm (A_550_). Data are shown as means ± standard deviation (SD). Statistically significant differences between groups were evaluated using Tukey’s test. *P < 0.05. n = 9/group.

**Fig. 5:**
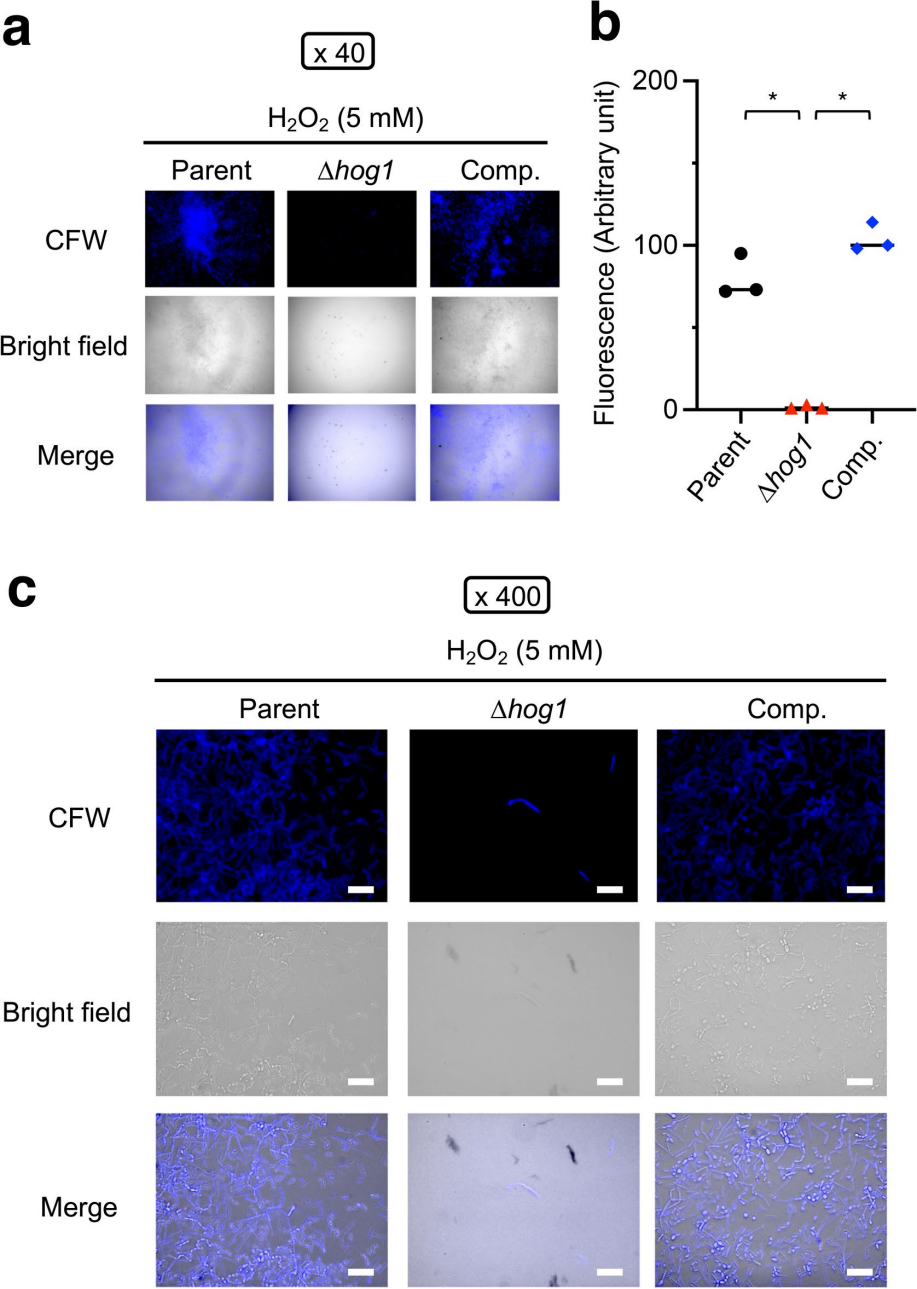
Effect of H_2_O_2_ on biofilm formation by the *hog1* gene-deficient mutant of *T. asahii* in vitro. Morphology of *T. asahii* stained with CFW was observed using fluorescence microscopy. The parent strain (Parent), the *hog1* gene-deficient mutant (∆*hog1*), and the complemented strain of ∆*hog1* (Comp.) in the biofilm were observed. CFW: calcofluor white stain. (**a**) Observed under a microscope at 40-fold magnification (×40). (**b**) Measurement of CFW fluorescence. Statistically significant differences between groups were evaluated using Tukey’s test. *P < 0.05. n = 3/group. (**c**) Observed under a microscope at 400-fold magnification (×400). White scale bar = 20 µm.

**Fig. 6:**
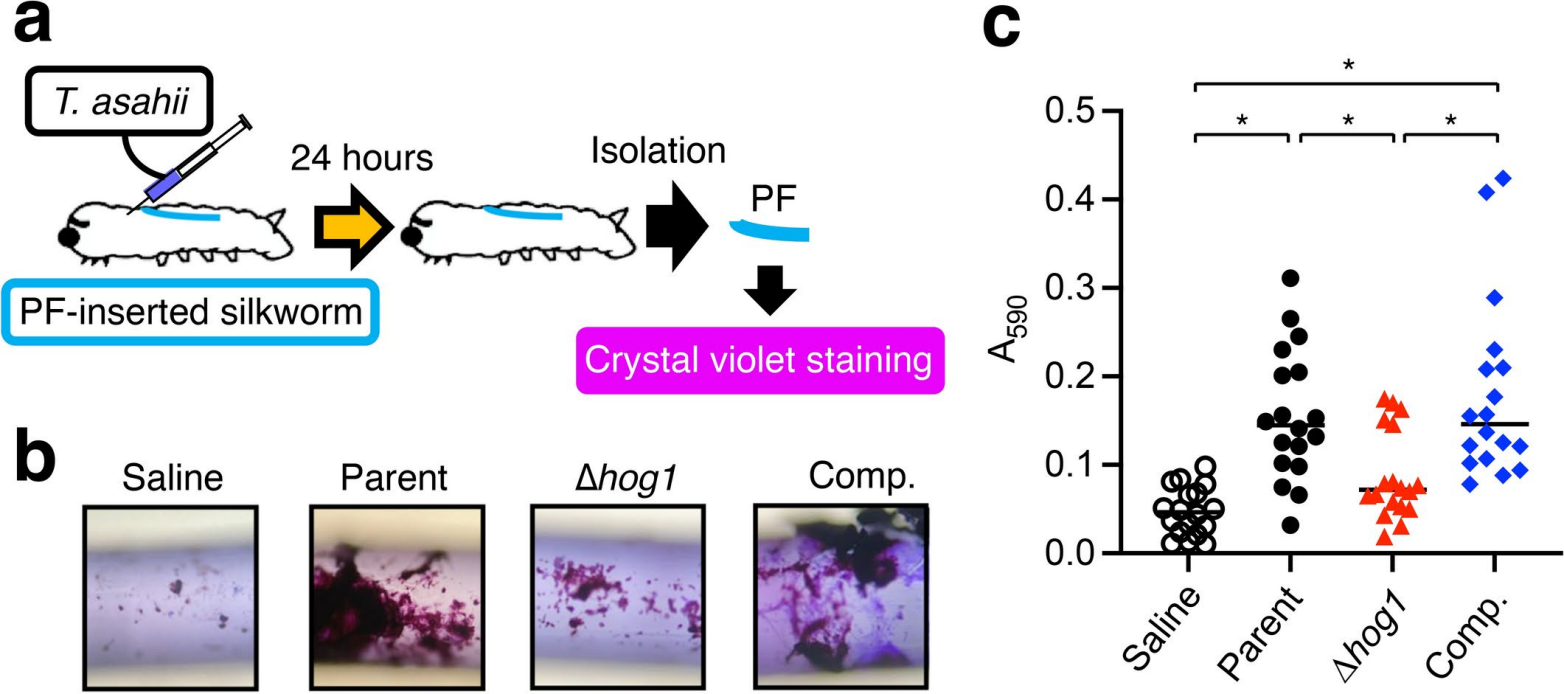
Effect of *hog1* gene-deficiency on *T. asahii* biofilm formation in vivo. (**a**) Experimental scheme of the in vivo biofilm assay using silkworms. Polyurethane fiber (PF)-inserted silkworms were prepared. Cell suspensions (A_630_ = 2) (50 µL) of the parent strain (Parent), the *hog1* gene-deficient mutant (∆*hog1*), and the complemented strain of ∆*hog1* (Comp.) were injected into the PF-inserted silkworms. The PF-inserted silkworms were incubated at 27 °C for 24 h. After incubation, PFs were isolated from the silkworms, stained with crystal violet, and observed under a microscope (**b**). The absorbance of the eluted dye was measured at 590 nm (**c**). Statistically significant differences between groups were evaluated using Tukey’s test. *P < 0.05. n = 18/group.

All experiments were performed at least triplicates, and representative results are shown. The significance of differences between multiple groups in Fig. 1b,d, 2a,b, 3b, 4b, 5b and 6c, was assessed using Tukey’s test. Statistical significance was set at *P* < 0.05.

## Results

### Hog1 negatively regulates *T. asahii* biofilm formation in vitro

In *C. albicans*, hyphal elongation is involved in biofilm formation^25^. The ratio of hyphae in biofilm produced by the *hog1* gene-deficient *T. asahii* mutant was increased in Sabouraud medium in vitro^13^. We examined whether the *hog1* gene affects biofilm formation by *T. asahii* in vitro. The amount of biofilm produced by the *hog1* gene-deficient *T. asahii* mutant in Sabouraud dextrose medium was increased compared with that of the parent strain (Fig. 1a,b). The *hog1* gene-deficient *T. asahii* mutant also exhibited higher biofilm mass in the RPMI medium than the parent strain (Fig. 1c,d). These phenotypes of the complemented strain were similar to those of the parent strain (Fig. 1). These results suggest that Hog1 negatively regulates biofilm formation by *T. asahii* in in vitro systems.

### *hog1* gene deficiency enhances cell viability in *T. asahii* biofilm

Biofilm comprises cells and an extracellular matrix of polysaccharides, proteins, nucleic acids, and lipids^30,31^. Because the *hog1* gene-deficient *T. asahii* mutant produced a large amount of biofilm, either the number of fungal cells in the biofilm or the amount of extracellular matrix might be increased. If the cell amount of the *hog1* gene-deficient mutant in the biofilm was increased, the cell viability would have been increased. Next, we examined the effect of the *hog1* gene deficiency on cell viability in the *T. asahii* biofilm. The cell viability in the biofilm produced by the *hog1* gene-deficient mutant was increased compared with that of the parent strain in Sabouraud dextrose medium and RPMI medium (Fig. 2). These phenotypes of the complemented strain were similar to those of the parent strain (Fig. 2). These results suggest *hog1* gene deficiency leads to an increased cell viability in the biofilm.

### *hog1* gene-deficiency causes increased hyphal formation in the biofilm in vitro

The cell viability of the *hog1* gene-deficient mutant in biofilm measured by the XTT assay was increased. Moreover, the ratio of hyphae in the biofilm produced by *hog1* gene-deficient mutant of *T. asahii* was increased in Sabouraud dextrose medium in vitro^13^. We hypothesized that hyphal formation was increased in the biofilm produced by the *hog1* gene-deficient mutant. *T. asahii* cells were stained with CFW, which binds to chitin in the cell wall. The *hog1* gene-deficient mutant formed long hyphae compared with the parent and complemented strains (Fig. 3). This finding suggests that the *hog1* gene is required for inhibiting the hyphal formation of *T. asahii* in biofilm.

### Oxidative stress inhibits biofilm formation by the *hog1* gene-deficient mutant

The *hog1* gene has an important role in the resistance of *T. asahii* to oxidative stress^13^. We investigated whether oxidative stress affects biofilm formation by the *hog1* gene-deficient *T. asahii* mutant. Under conditions of oxidative stress induced by H_2_O_2_, biofilm formation by the *hog1* gene-deficient mutant was lower than that by the parent and complemented strains (Fig. 4). Long hyphae were not observed in the biofilm produced by the *hog1* gene-deficient mutant (Fig. 5). These observations suggest that biofilm formation by the *hog1* gene-deficient mutant was inhibited by oxidative stress.

### Essential role of the *hog1* gene in biofilm formation by *T. asahii* in an in vivo system using silkworms

*T. asahii* is subjected to various stressors, including oxidative stress, in the host environment during infection^32^. An in vivo system using silkworms was established for evaluating biofilm formation by *C. albicans*^23^. Using this method, we examined whether the *hog1* gene is involved in biofilm formation by *T. asahii* in vivo. Biofilm formation by the *hog1* gene-deficient mutant was decreased compared with that by the parent strain (Fig. 6). The phenotype of the complemented strain was similar to that of the parent strain (Fig. 6). These results suggest that the *hog1* gene is required for biofilm formation by *T. asahii* in an in vivo system using silkworms.

## Discussion

In the present study, we examined the role of the *hog1* gene in biofilm formation by *T. asahii* in vitro and in vivo. In an in vitro experiment without oxidative stress, the *hog1* gene negatively regulated the biofilm formation and transition to hyphae. On the other hand, an in vitro experiment with oxidative stress demonstrated that the *hog1* gene was required for biofilm formation. Moreover, in an in vivo experiment using silkworms, the *hog1* gene was required for biofilm formation by *T. asahii* on the surface of a polyurethane fiber in silkworms. These results suggest that Hog1-mediated stress tolerance plays an important role in biofilm formation in the host environment, including under conditions of oxidative stress.

**Fig. 7:**
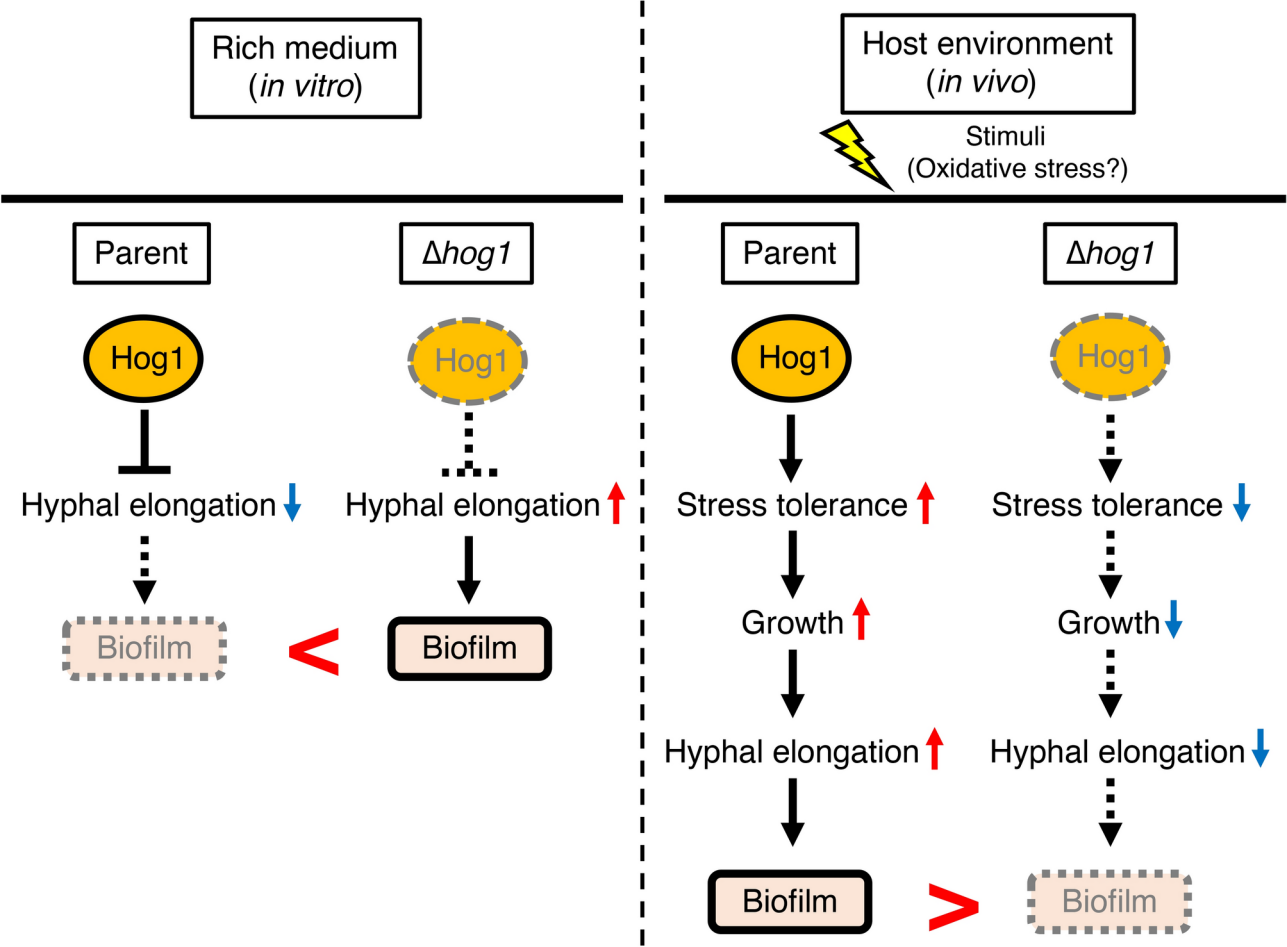
Model of the role of Hog1 in biofilm formation by *T. asahii* according to environmental conditions. Under rich medium conditions in vitro, Hog1 negatively regulates hyphal elongation and biofilm formation. In host environments in vivo, *T. asahii* cells are subjected to several stimuli including oxidative stress, and Hog1-mediated stress tolerance is required for biofilm formation.

Hog1 negatively regulates biofilm and hyphae formation in biofilm under rich medium conditions. Gene deficiency of *hog1* leads to excess biofilm formation by *T. asahii* under rich medium conditions such as Sabouraud dextrose medium and RPMI medium. The biofilm mass by *T. asahii* parent strain was higher on RPMI medium than on Sabouraud dextrose medium. We assumed that the RPMI medium was richer in vitamins and salts under neutral conditions than the Sabouraud dextrose medium, and this may have contributed to the better growth of *T. asahii* in the biofilm. Biofilm is formed by microorganisms, which secrete molecules such as proteins, DNA, and polysaccharides^30,31^. Therefore, we assumed that biofilm produced by the *hog1* gene-deficient *T. asahii* mutant contained a large number of cells and/or large amounts of extracellular matrix. The cell viability of the *hog1* gene-deficient mutant in biofilm was increased. Moreover, the difference in the cell viability per biofilm mass values between parent strain and the *hog1* gene-deficient *T. asahii* mutant was less than twofold (Supplementary Fig. S1). This finding suggests the presence of a large number of cells or a large cell in the biofilm formed by the *hog1* gene-deficient mutant. The proportion of hyphae in the *hog1* gene-deficient mutant grown in Sabouraud dextrose medium was increased^13^. In biofilm formed by *C. albicans*, hyphae are observed^33^. Therefore, we hypothesized that the hyphal formation of the *hog1* gene-deficient mutant was involved in the excess biofilm formation by *T. asahii* under rich medium conditions. In a biofilm, the *hog1* gene-deficient mutant showed long hyphal formation. This observation suggests that the hyphal formation of the *hog1* gene-deficient mutant is a key factor in the excess biofilm formation. We concluded that Hog1 negatively regulates biofilm formation by inhibiting hyphal formation in the biofilm under rich medium conditions (Fig. 7).

Hog1 is required for biofilm formation by *T. asahii* under oxidative stress conditions. Fungal pathogens, including *T. asahii,* are exposed to oxidative stress during bloodstream infections^33^. Therefore, oxidative stress affects biofilm formation by *T. asahii* on catheter surfaces in the human bloodstream. The *hog1* gene-deficient mutant was sensitive to oxidative stress such as that induced by H_2_O_2_^13^. Biofilm formation by *T. asahii* under oxidative stress conditions was reduced by *hog1* gene deficiency. This result suggests that *hog1* is required for biofilm formation by *T. asahii* under oxidative stress conditions. We concluded that Hog1-mediated stress tolerance has a crucial role in biofilm formation by *T. asahii* under oxidative stress in vitro.

Hog1-mediated stress tolerance is required for biofilm formation by *T. asahii* in an in vivo system using silkworms. A silkworm infection model was established to evaluate the virulence of *T. asahii*^34^. In the silkworm infection model, the virulence of *T. asahii* was decreased by the *hog1* gene deficiency^13^, indicating that *hog1* is required for *T. asahii*^13^. An in vivo system using silkworms for measuring the biofilm formation by *C. albicans* was established^23^. In this in vivo system using silkworms, we evaluated biofilm formation by *T. asahii* in vivo and found that the *hog1* gene is required for biofilm formation by *T. asahii*. Therefore, we assumed that Hog1-mediated stress tolerance plays an important role in *T. asahii* biofilm formation in vivo (Fig. 7). Moreover, we performed the effect of human serum on biofilm formation by *T. asahii* in vitro (Supplementary Fig. S2). The biofilm mass of *T. asahii* in human serum was lower than that in Sabouraud dextrose medium and RPMI medium (Supplementary Fig. S2). Human serum contains several compounds including antimicrobial peptides^35^. We assumed that several host factors in human serum affect biofilm formation by *T. asahii*. The identification of in vivo stressors that induce Hog1-mediated stress tolerance is an important issue.

In *C. albicans*, the *hog1* gene deficiency causes excess hyphal formation^36^. Moreover, the *hog1* gene-deficient mutant of *C. albicans* showed sensitivity to several stressors including oxidative stress^15^. In *T. asahii*, Hog1 regulates hyphal formation and oxidative stress tolerance^13^. Therefore, the functions of Hog1-mediated stress tolerance were conserved between *C. albicans* and *T. asahii*. We assumed that Hog1 was also required for biofilm formation by *C. albicans* in vivo. Future studies on the effect of Hog1 on in vivo biofilm formation in pathogenic fungi other than *T. asahii* are needed.

Fungal specific-Hog1 inhibitors may attenuate biofilm formation by *T. asahii* on a catheter surface during bloodstream infection. Although calcineurin is widely conserved in eukaryotes like Hog1, a fungal-specific calcineurin inhibitor has been developed based on structural differences between human calcineurin and fungal calcineurin^37^. Therefore, a protein structure-based drug design approach may contribute to the development of fungal-specific Hog1 inhibitors. We speculate that fungal specific-Hog1 inhibitors could reduce *T. asahii* biofilm formation during catheter-related chronic infections. The development of fungal-specific Hog1 inhibitors will be a focus of future research.

## Conclusion

Hog1-mediated stress tolerance plays an important role in biofilm formation by *T. asahii* through adaptation to the host environment. Hog1 is a potential target for anti-biofilm drugs. In vivo evaluation is essential for understanding biofilm formation by pathogens during infections.

## Supplementary Information

Supplementary Information 1.

Supplementary Figures.

## Data Availability

All data generated or analyzed during this study are included in this published article and its Supplementary Information file.
