# Supplementary material for: Role of Hog1-mediated stress tolerance in biofilm formation by the pathogenic fungus Trichosporon asahii
Source: Sci Rep. 2024 Nov 20;14:28761. doi: 10.1038/s41598-024-80200-z (PMC11579369; doi:10.1038/s41598-024-80200-z)
Supplement: Supplementary file 2 — Supplementary Figures. [file 41598_2024_80200_MOESM2_ESM.docx]

**Supplementary information**

**Role of Hog1-mediated stress tolerance in biofilm formation by the pathogenic fungus *Trichosporon asahii***

**Yasuhiko Matsumoto^1*^, Mei Nakayama^1^, Yuta Shimizu^1^, Sachi Koganesawa^1^, Hiromi Kanai^1^, Yu Sugiyama^1^, Sanae Kurakado^1^, and Takashi Sugita^1^**

^1^Department of Microbiology, Meiji Pharmaceutical University, 2-522-1, Noshio, Kiyose, Tokyo 204-8588, Japan.

*Address correspondence to: Dr. Yasuhiko Matsumoto, Department of Microbiology, Meiji Pharmaceutical University, 2-522-1, Noshio, Kiyose, Tokyo 204-8588, Japan, Tel: +81-42-495-8745, e-mail: [ymatsumoto@my-pharm.ac.jp](mailto:ymatsumoto@my-pharm.ac.jp).


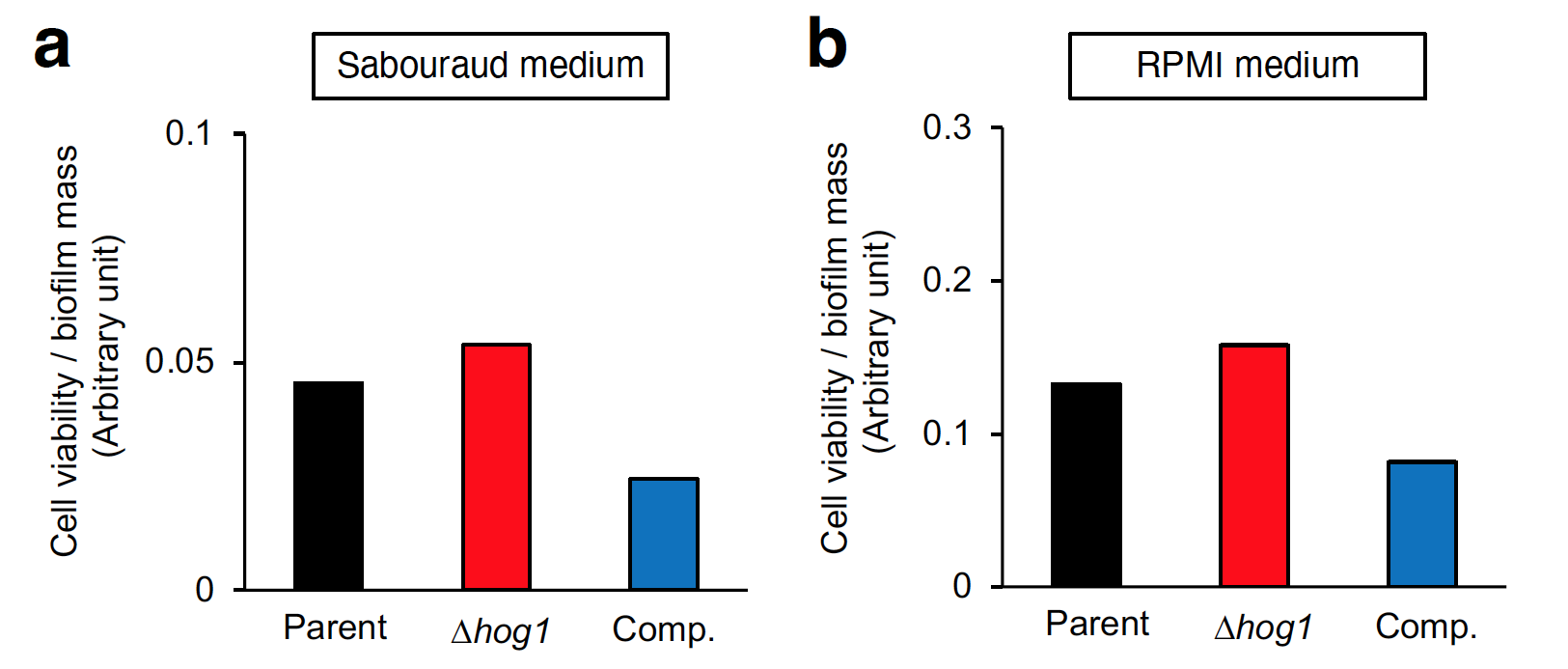


**Supplementary Fig. S1** Cell viability per biofilm mass value was determined from the data shown in Figures 1 and 2. (**a**) Sabouraud dextrose medium. (**b**) RPMI medium.


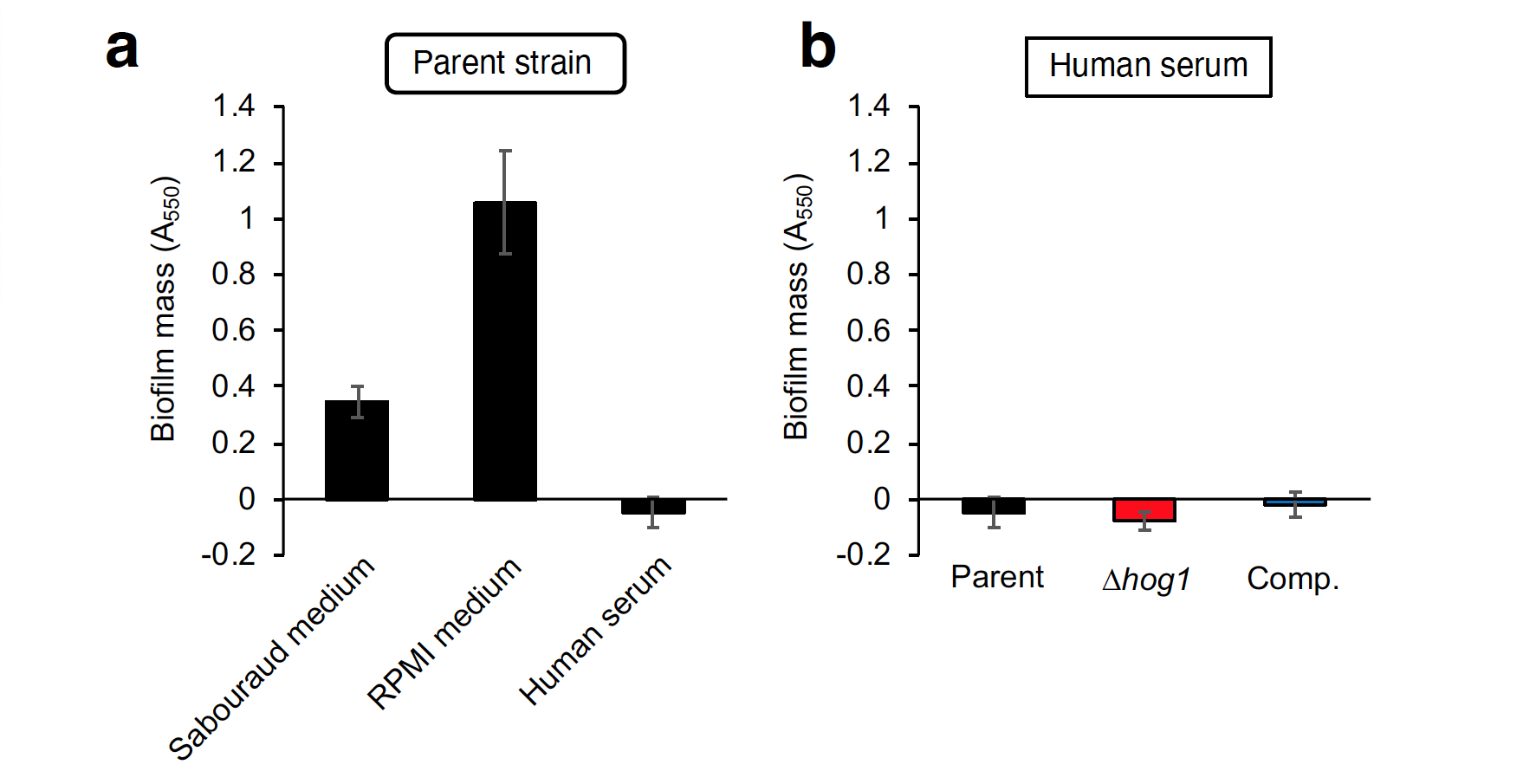


**Supplementary Fig. S2** Effects of medium to biofilm formation by *T. asahii in vitro*. (a) Biofilm formation by *T. asahii* in Sabouraud dextrose medium, RPMI medium, or human serum *in vitro* was determined by crystal violet (CV) staining. n = 3-5/group. (b) Biofilm formation by *T. asahii* in RPMI medium *in vitro*. The amounts of biofilm formation by the parent strain (Parent), *hog1* gene-deficient mutant (∆*hog1*), and its complement strain (Comp.) in the RPMI medium were determined by CV staining. n = 3/group.
